# Handedness effects on motor imagery during kinesthetic and visual-motor conditions

**DOI:** 10.1038/s41598-021-92467-7

**Published:** 2021-06-23

**Authors:** Dariusz Zapała, Paulina Iwanowicz, Piotr Francuz, Paweł Augustynowicz

**Affiliations:** Department of Experimental Psychology, The John Paul II Catholic University of Lublin, Al. Racławickie 14, 20-950 Lublin, Poland

**Keywords:** Neuroscience, Psychology

## Abstract

Recent studies show that during a simple movement imagery task, the power of sensorimotor rhythms differs according to handedness. However, the effects of motor imagery perspectives on these differences have not been investigated yet. Our study aimed to check how handedness impacts the activity of alpha (8–13 Hz) and beta (15–30 Hz) oscillations during creating a kinesthetic (KMI) or visual-motor (VMI) representation of movement. Forty subjects (20 right-handed and 20 left-handed) who participated in the experiment were tasked with imagining sequential finger movement from a visual or kinesthetic perspective. Both the electroencephalographic (EEG) activity and behavioral correctness of the imagery task performance were measured. After the registration, we used independent component analysis (ICA) on EEG data to localize visual- and motor-related EEG sources of activity shared by both motor imagery conditions. Significant differences were obtained in the visual cortex (the occipital ICs cluster) and the right motor-related area (right parietal ICs cluster). In comparison to right-handers who, regardless of the task, demonstrated the same pattern in the visual area, left-handers obtained higher power in the alpha waves in the VMI task and better performance in this condition. On the other hand, only the right-handed showed different patterns in the alpha waves in the right motor cortex during the KMI condition. The results indicate that left-handers imagine movement differently than right-handers, focusing on visual experience. This provides new empirical evidence on the influence of movement preferences on imagery processes and has possible future implications for research in the area of neurorehabilitation and motor imagery-based brain–computer interfaces (MI-BCIs).

## Introduction

Mental imagery of movement (or motor imagery, MI) can be defined as “the mental representation of action without any concomitant body movement”^1^ (p. 31). However, the nature of representations forming mental images was intensively discussed in the last decades of the twentieth century and at the beginning of the twenty-first century. We know that mental representations have a heterogeneous nature which is closely related to the type of information being processed^2^. When it comes to the creation of a mental representation of a given movement, the subject may imagine seeing himself or another person acting (exterior view from the third-person perspective) or a self-performed action that is accompanied by the feeling of actually performing the task (kinesthetic experience from the first-person perspective)^3^. Kinesthetic representation (or kinesthetic motor imagery, KMI) may include somesthetic sensations associated with self-movement and body position. On the other hand, visual representations of action (or visual-motor imagery, VMI) are similar to visual imagery but concern moving objects. Guillot et al.^4^ compared the MI in kinesthetic and visual conditions and reported that VMI activated predominantly the occipital regions (BA17-19) and the superior parietal lobules (BA5, 7), whereas KMI yielded more activity in motor-associated structures (BA6, 44) and the inferior parietal lobule (BA40). In another fMRI study, Kilintari et al.^5^ demonstrated the difference between the brain activation patterns sustaining the process of imagining using VMI or KMI, that is the degree of deactivation of visual areas (BA17, 18, 19, 37). Also, studies using TMS confirm separation of both types of representations at the cortical activity level. Stainer et al.^6^ reported that the primary motor cortex (M1) is modulated only in the KMI condition, while Mizuguchi, Nakamura, and Kanosue^7^ show that visual, but not kinesthetic MI increase excitability in the primary visual cortex.

In the EEG/MEG domain, it has been observed that kinesthetic and visual-motor images of movement evoke different alpha/mu and beta wave distribution patterns, which affect e.g., the classification of the signal in the brain-computer interfaces (BCIs)^8^. During kinesthetic MI, the event-related desynchronization (ERD) of the sensorimotor rhythms (SMR) is more strongly lateralized, like in real movement. In the case of VMI, the lateralization effect is less visible, and the pattern of activity is similar to movement observation^3^. Igasaki, Takemoto, and Sakamoto^9^ also showed that kinesthetic and visual MI differentiate alpha-ERD in the occipital area, depending on how difficult each task was for the subject. For those who thought it was easy for them to imagine movement, differences in alpha power per O1 and O2 electrode between KMI and VMI conditions were observed. Also, in the study by Neuper et al.^3^, it has been shown that alpha and beta waves in the occipital region are the most important features in identifying VMI condition. Moreover, Bagherzadeh et al.^10^ argue that synchronisation in the occipital alpha band corresponds to inhibition of visual cortex activity and may be modulated by intentional attention orientation changes. Therefore, both sensorimotor and visual areas should be considered in studying EEG activity associated with motor imagery.

Furthermore, there are intra- and interindividual differences in the abilities and preferences to imagine movement in a kinesthetic and visual-motor way. Some studies show that people who prefer KMI rather than VMI perspective also achieve better performance in Motor Imagery-BCI control (MI-BCI)^11^. Although the use of self-description questionnaires to predict the classification of the EEG signal could be controversial^12^. Other studies have shown that the choice of a motor imagery perspective may arise from individual differences, e.g., handedness. Using the *hand laterality task* (HLT), Ní Choisdealbha et al.^13^ have noticed that the time when right- and left-handed people assess the position of a hand displayed on the screen depending on the current position of their own hands differs. It was noted that left-handed individuals could rely more on the hand position's visual assessment than on sensorimotor sensations such as the right-handed. Overall, while performing a mental rotation of hands, shorter execution times were observed in right-handed people, whether the task involved their dominant or non-dominant hand^14^. A potential source of these variations may lie in different cortical organisations and brain networks dynamics. In TMS study, Nicolini et al.^15^ found that left-handers show more symmetry of the upper limb muscles' cortical representation but a smaller spatial range of this representation than right-handers. Less asymmetry of the motor system in left-handers was also observed in resting-state fMRI effective connectivity study^16^. A similar pattern in differences is present not only to the motor areas but also to the activations of visual areas related to face and body perception^17^.

In their case study, Carino-Escobar et al.^18^ were looking to verify how these factors affect MI-BCI performance by analyzing physiological, anatomical, and psychological differences between monozygotic twins discordant handedness. Contrary to the authors' expectations, the left-handed participant achieved better BCI control, whether the feedback was provided visually or kinesthetically. The opposite effect was observed in the group study^19^ where EEG activity patterns and MI-BCI control in left and right-handers were compared. In this case, better MI-BCI performance and alpha-ERD lateralization were reported in the right-handed group.

The experiments described above have shown that handedness can affect the mental representation of movement, reflected both at the behavioral level^13^ and in differences in brain activity patterns^18,19^. Casasanto^20^ suggests that due to different physical interaction experiences with the environment, right- and left-handed people have also developed other understanding of the same abstract concepts. Therefore, discrepancies in previous BCI experiments' results may indicate additional factors influencing the differences between right and left-handers. For instance, providing imagery instructions in an explicit or implicit form has a significant impact on MI's neuronal correlates. In the study of Mizuguchi, Suezawa, and Kanosue' studies^21^ it was found that the performance of the visual-motor imagery task (sequential finger movement) correlates with the activity of the areas of the visual cortex, while subjective assessment of the vividness of these images with the orbitofrontal cortex. However, many MI experimental procedures do not contain any behavioral measures of performance^22^, or instructions indicating the motor imagery perspective (e.g., KMI or VMI). Also, in the experiment by Zapała et al.^19^, there was no control on how participants perform imagery movements. Whereas in Carino-Escobar et al.^18^ study, the subjects could use different imagining movement strategies, whether the feedback was given visually or somatosensorily. Because of these limitations of previous research, the observed opposite results may be caused by a lack of control of the MI performance strategies.

This study aims to verify the effect of handedness on brain waves activity and the correctness of creating kinesthetic and visual-motor representations of movement. We used independent component analysis (ICA) decomposition to identify the motor-related and visual-related neural substrates of brain activity evoked by the KMI and VMI perspectives. The scope of analyses was limited to the alpha (8–13 Hz) and beta (15–30 Hz) bands corresponding with the range of sensorimotor rhythms in the motor cortex^23^ and visual cortex activity^24^ (Supplementary Table 1).

We hypothesized that handedness would influence both electroencephalographic and behavioural correlates of motor imagery. According to previous studies^13^, right-handers will more easily take the KMI perspective than VMI and the opposite for left-handers. Therefore we assume that the correctness of KMI condition will be higher in the right-handed group and the VMI condition in the left-handed group. Moreover, in the KMI condition, the desynchronization in the SMR on the side opposite to the hand involved in the MI task will be higher in the group of right-handed people. As in the previous EEG^19^, fMRI^16^ or TMS^15^ studies, we suppose that motor cortex activity's asymmetry will be more pronounced in right-handers. When it comes to the VMI condition, the increase of power in the alpha and beta range will be greater in the group of left-handed participants. Such a result would correspond to the visual cortex's stronger deactivation during the VMI condition, observed in other fMRI studies^5^.

## Results

### EMG activity

There were no main effects or interactions of the factors Group *F*(1,31) = 1.6, *p* = 0.215; Task *F* (1,31) = 2.36, *p* = 0.135, and Hand *F* (1,31) = 3.18, *p* = 0.09, which indicate similar muscle tension in both groups and all experimental conditions. Significant differences were observed only in the Channel factor *F* (1,31) = 4.33, *p* = 0.045, *n*_*p*_^*2*^ = 0.12. The electrode placed on the left forearm registered higher amplitude than the one that was placed on the right forearm (Channel-left *M* = 0.14 mV, *SE* = 0.08 mV; Channel-right *M* = 0.04 mV, *SE* = 0.03 mV) (Supplementary Fig. 1).

### Behavioral performance

**Figure 1:**
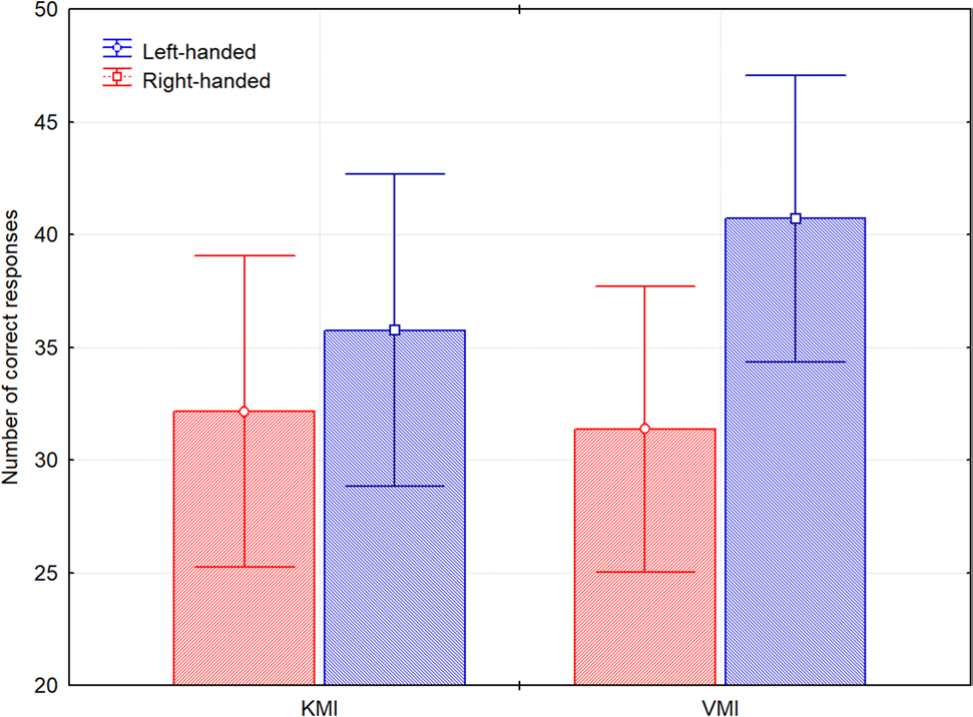
Differences in the number of correct responses related to the effects of Task × Group. The vertical bars represent standard error. Used software to create the images in this figure: STATISTICA version 12 (StatSoft, Inc., Tulsa, OK, USA, https://www.tibco.com/).

The main effect of the factor Group was significant, *F*(1,38) = 4.44, *p* = 0.04, *n*_*p*_^*2*^ = 0.10, indicating that the correctness (number of correct answers) was higher in the left-handed group (right-handed: *M* = 31.76, *SE* = 2.17; left-handed: *M* = 38.22, *SE* = 2.17). In addition, we found the significant interaction of the factors Task × Group, *F* (1,38) = 6.14, *p* = 0.018, *n*_*p*_^*2*^ = 0.14, and the Bonferroni post hoc test revealed (*p* = 0.02), that the left-handers performed the VMI task better (*M* = 40.7, *SE* = 2.21) than the KMI task (*M* = 35.75, *SE* = 2.41). Also, in the VMI condition the left-handed subjects achieved significantly (*p* = 0.038) more correct answers (*M* = 40.7, *SE* = 2.21) than right-handers (*M* = 31.37, *SE* = 2.21) (Fig. 1). There were no main effects or interactions of the factors Task *F* (1,38) = 3.26, *p* = 0.078, and Hand *F* (1,38) = 2.79, *p* = 0.1.

### EEG activity

**Figure 2:**
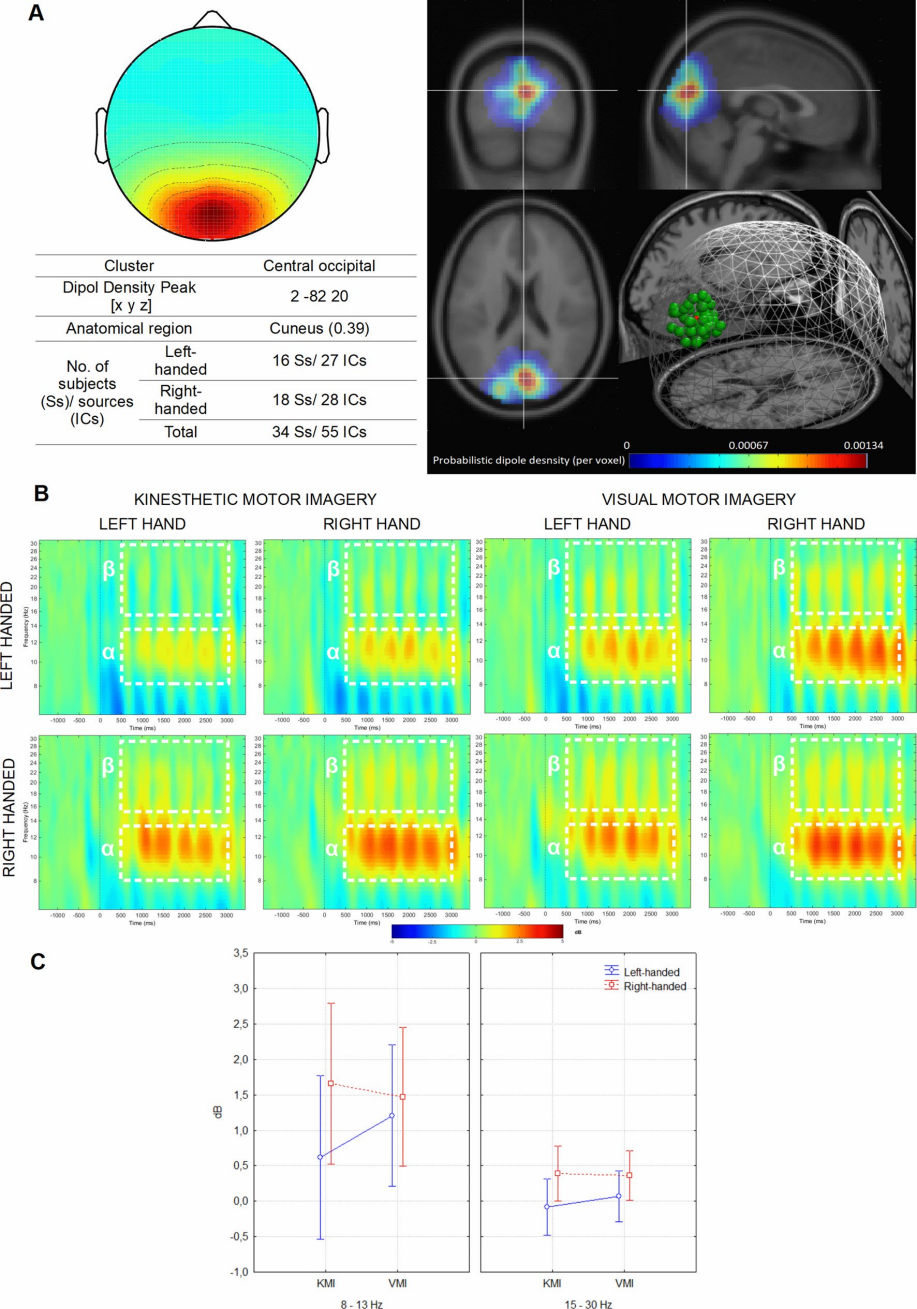
Central occipital cluster. (**A**) Average topography with the estimated anatomical location of the centroid (top left) and visualization of probabilistic dipoles density (top right); (**B**) mean ERSP time × frequency spectrograms from all components for both groups during left- and right hand MI in the KMI and VMI conditions. White dotted lines indicate alpha (α) and beta (β) time–frequency regions averaged for the statistical analysis. (**C**) Differences in alpha and beta band power related to the effects of Frequency × Task × Group. The vertical bars represent standard error. Used software to create the images in this figure: MATLAB version R2014b (MathWorks, Natick, MA, USA, https://www.mathworks.com/).

**Figure 3:**
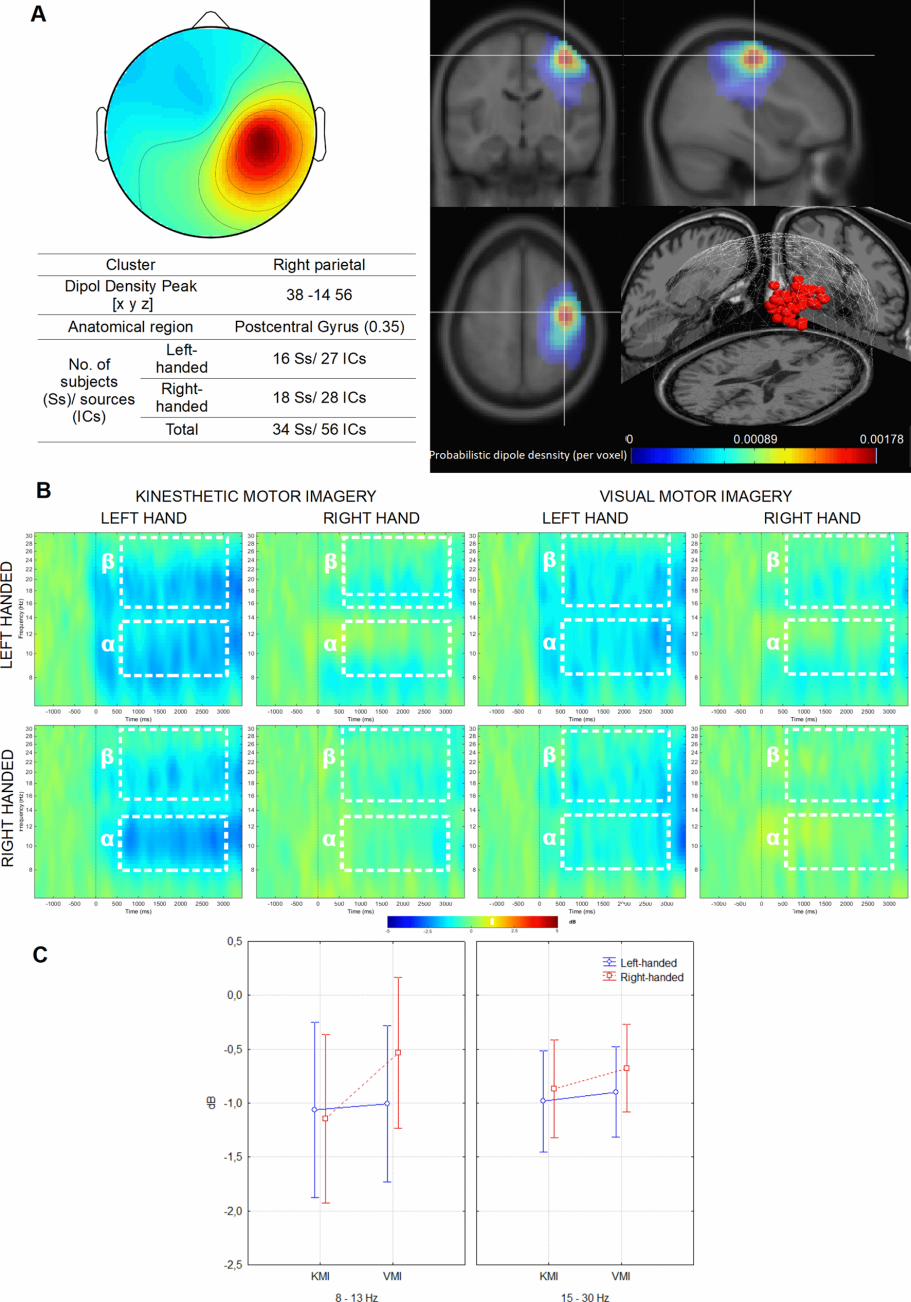
Right parietal cluster. (**A**) Average topography with the estimated anatomical location of the centroid (top left) and visualization of probabilistic dipoles density (top right); (**B**) mean ERSP time × frequency spectrograms from all components for both groups during left- and right hand MI in the KMI and VMI conditions. White dotted lines indicate alpha (α) and beta (β) time–frequency regions averaged for the statistical analysis. (**C**) Differences in alpha and beta ERD related to the effects of Frequency × Task × Group. The vertical bars represent standard error. Used software to create the images in this figure: MATLAB version R2014b (MathWorks, Natick, MA, USA, https://www.mathworks.com/).

**Figure 4:**
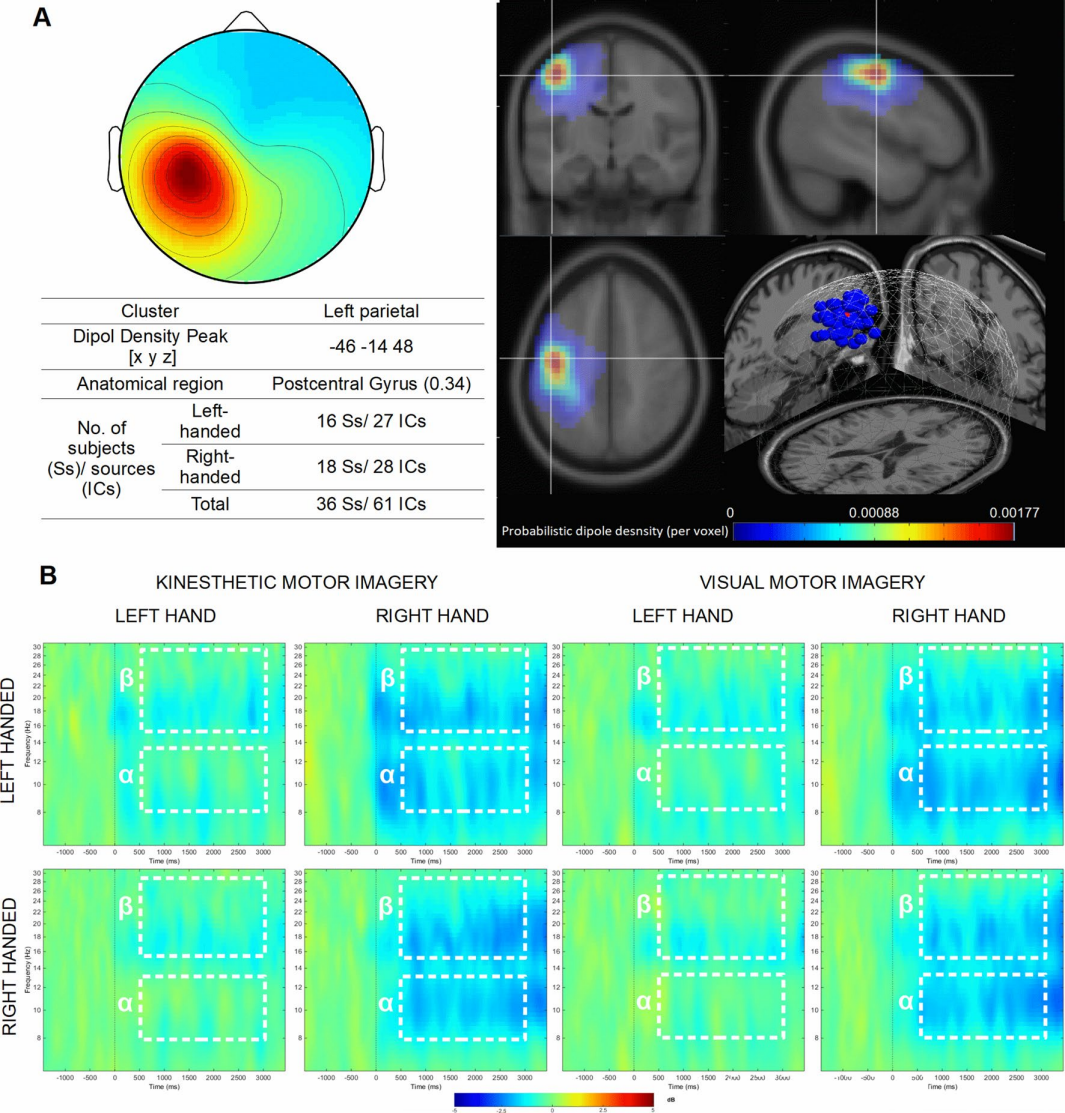
Left parietal cluster. (**A**) Average topography with the estimated anatomical location of the centroid (top left) and visualization of probabilistic dipoles density (top right); (**B**) mean ERSP time × frequency spectrograms from all components for both groups during left- and right hand MI in the KMI and VMI conditions. White dotted lines indicate alpha (α) and beta (β) time–frequency regions averaged for the statistical analysis. Used software to create the images in this figure: MATLAB version R2014b (MathWorks, Natick, MA, USA, https://www.mathworks.com/).

After preprocessing of EEG data 749 ICs were obtained and have been classified using the *k*-means (*k* = 15) algorithm into 15 clusters based on dipole locations (Supplementary Fig. 3), scalp projection maps, and signal strength (8–30 Hz). Next, anatomical regions were estimated for each cluster based on dipole density. The two of them (Cls 4 and Cls 11) were right and left parietal clusters with source estimated in the right and left postcentral gyrus (Supplementary Fig. 2). These clusters showed stronger desynchronization of alpha and beta frequencies contralateral to the hand involved in the imagery task (Figs. 3B and 4B). In the case of central occipital Cls 10, the signal source has been located in *cuneus* (Fig. 2B), a part of the occipital cortex involved in visual processing^25^. Therefore, the right/left parietal and central occipital clusters were selected for analysis as representing activities from the motor or visual-related cortex.

#### Central occipital cluster

There were no main effects of the factors Group *F*(1,31) = 2.51, *p* = 0.119, and Task *F* (1,31) = 3.37, *p* = 0.068. However, we have noted significant main effects for factors Frequency *F* (1,53) = 24.57; *p* < 0.001, *n*_*p*_^*2*^ = 0.32, and Hand *F*(1,53) = 6.04; *p* = 0.017, *n*_*p*_^*2*^ = 0.08. The alpha frequency (*M* = 1.24 dB, *SE* = 0.26 dB) had higher strength than beta (*M* = 0.18 dB, *SE* = 0.09 dB). Stronger signal was also recorded during the imaginary movement of the right hand (Left: *M* = 0.61 dB, *SE* = 0.16 dB; Right: *M* = 0.81 dB, *SE* = 0.17 dB). Moreover, we observed a significant Task × Group interaction *F*(1,53) = 11.52, *p* = 0.001, *n*_*p*_^*2*^ = 0.18. The Bonferroni post hoc test showed that a significantly (*p* = 0.003) stronger signal was recorded during the VMI task in the left-handed group (KMI: *M* = 0.27 dB, *SE* = 0.26 dB ; VMI: *M* = 0.64 dB, *SE* = 0.22 dB). The results of the interaction between factors Frequency × Task × Group was also significant *F*(1,53) = 9.16, *p* = 0.004, *n*_*p*_^*2*^ = 0.15, and post hoc comparisons made it clear that the difference between the KMI and VMI tasks is present only in the left-handed group (*p* < 0.001), for the 8–13 Hz frequency band (KMI: *M* = 0.62 dB, *SE* = 0.41 dB; VMI: *M* = 1.2 dB, *SE* = 0.35 dB) (Fig. 2).

#### Right parietal cluster

There were no main effects of the factors Group *F*(1,54) = 0.49; *p* = 0.515, and Frequency *F*(1,54) = 0.429; *p* = 0.515. However, we have noted significant main effects for factors Task *F*(1,54) = 5.19; *p* = 0.026, *n*_*p*_^*2*^ = 0.09, and Hand *F*(1,54) = 56.65; *p* < 0.001, *n*_*p*_^*2*^ = 0.51. Desynchronization was greater in the KMI condition (KMI: *M* = − 1.01 dB, *SE* = 0.15 dB; VMI: *M* = − 0.77 dB, *SE* = 0.13 dB), and during the motor imagery of the left hand (Left: *M* = − 1.33 dB, *SE* = 0.16 dB; Right: *M* = − 0.47 dB, *SE* = 0.12 dB). Moreover, we observed a significant Frequency × Hand interaction *F*(1,54) = 15.04, *p* < 0.001, *n*_*p*_^*2*^ = 0.21. The Bonferroni post hoc test showed a significantly (*p* < 0.001) stronger suppression in the alpha frequency band, during the motor imagery of the left hand (8–13 Hz: *M* = − 1.49 dB, *SE* = 0.23 dB; 15–30 Hz: *M* = − 1.15, *SE* = 0.12 dB). The results of the interaction between factors Frequency × Task × Group were also significant *F*(1,54) = 9.16, *p* = 0.004, *n*_*p*_^*2*^ = 0.15, and post hoc comparisons showed that the difference between the KMI and VMI tasks is present only in the right-handed group (*p* < 0.001), for the 8–13 Hz frequency band (KMI: *M* = − 1.14 dB, *SE* = 0.27 dB; VMI: *M* = − 0.53 dB, *SE* = 0.25 dB) (Fig. 3).

#### Left parietal cluster

There were no main effects of the factors Group *F*(1,59) = 0.04; *p* = 0.832; Task *F*(1,59) = 0.01; *p* = 0.938, and Frequency *F*(1,59) = 0.267; *p* = 0.607. We have noted only one significant main effect for factor Hand *F*(1,59) = 48.55; *p* < 0.001, *n*_*p*_^*2*^ = 0.45, and Frequency × Hand interaction *F*(1,59) = 17.97, *p* < 0.001, *n*_*p*_^*2*^ = 0.23. Desynchronization was greater during the motor imagery of the right hand (Left: *M* = − 0.58 dB, *SE* = 0.14 dB; Right: *M* = − 1.45 dB, *SE* = 0.16 dB). Moreover, post hoc comparisons showed that during the left (*p* < 0.001), and right (*p* = 0.002) motor imagery of hand movement, suppression was stronger in alpha frequency band (Left_8–13 Hz_: *M* = − 0.47 dB, *SE* = 0.19 dB, _15–30 Hz_ M = − 0.69 dB, *SE* = 0.1 dB; Right_8–13 Hz_: *M* = − 1.63 dB, *SE* = 0.23 dB; _15–30 Hz_ M = − 1.27 dB, *SE* = 0.11 dB) (Fig. 4).

## Discussion

The results of the performed statistical analyses confirmed some of the research hypotheses. Indeed, the handedness significantly impacts the performance of imagery tasks. Similarly to the case study by Carino-Escobar et al.^18^, the left-handed achieved higher accuracy than the right-handed. However, the group differences of the KMI and VMI conditions matched the hypotheses only in the left-handers' case. In this group, as expected, the VMI task was performed significantly better than KMI and on a higher level than by the right-handed. At the same time, the opposite effect was not found in the second group, where there were no significant differences in correctness between the KMI and VMI conditions. Therefore, we cannot confirm that the right-handed prefer a kinesthetic perspective in motor imagery, and this is the reason for better control, e.g. in MI-BCIs^19^.

It is possible that this contradiction in the results is caused by the different relevance of the mental perspective for the functioning of individuals in both groups. Some researchers suggest that left-handed people achieve better coordination of their hands due to the environmental pressure of using objects designed for the right-handed^18^. For the same reason, the left-handed are forced to change the perspective of the perceived scene or object in their mind, so the VMI condition is more familiar to them. On the other hand, the right-handers rarely have to change the perspective of the seen objects in their minds, so they use visual-motor and kinesthetic imagery equally.

The results of the EEG activity analyses from the occipital region support the research hypothesis. As predicted, the left-handed subjects showed more robust synchronization in the alpha band in the VMI condition compared to the KMI. Similarly to the behavioural measurement, no significant results were observed among the right-handed. Activity in the alpha and beta band in the central occipital cluster did not differ significantly in the right-handers between experimental conditions. The power increase (synchronization) over occipital areas, especially in the alpha band, is linked to the engagement in visual-motor imagery of movement^3^. Studies on the occipital and parietal alpha rhythms show that the power increase may represent inhibition of visual cortex activity^10^. Therefore, in the VMI condition, alpha-ERS in the central occipital cluster may represent a shifting from processing external stimuli to evoking internal states. Also in the fMRI studies, Killari et al.^5^ noted that deactivation of visual areas may indicate better concentration on internal states and engagement of the default mode network (DMN). The effects observed over the central occipital areas may indicate that it is easier for left-handed subjects to imagine a movement from a VMI perspective than KMI. This may also indicate better general ability to perform the imagined movement. Igasaki, Takemoto, and Sakamoto^9^ showed that the occipital alpha suppression differences between the KMI and VMI conditions are higher for subjects who reported fewer difficulties in the motor imagery task. Therefore, the right-handed participants, who performed the MI tasks weakly, had no significant differences in alpha and beta activity in the occipital areas.

For the occipital cluster, regardless of the experimental condition and group, the registered power was significantly higher for the right hand MI and alpha band. According to some interpretations of oscillations from occipital areas^24^, alpha rhythms correspond to the visual cortex's basal excitation, while beta rhythms reflect changes in visual attention. It is possible that in the KMI and VMI conditions, visual attention was engaged in the same way on the displayed cues, but both types of imagery inhibited visual processing to a different degree. Therefore, the intra- and intergroup effects mentioned earlier could be interpreted as differences in attenuating external stimuli perception. Moreover, for right- and left-handed participants, imagining a right-hand movement could have induced similarly vivid mental representations and consequently more robust occipital area response.

The other effects were noted for desynchronization of sensorimotor rhythms in areas related to the left-hand movement (right parietal cluster). According to the hypothesis, the right-handed subjects showed higher suppression in the alpha (or mu) band in the KMI condition and during the MI of the contralateral hand. For the left-handers, the alpha and beta rhythms did not differ significantly between the imagery tasks. At the same time, the SMR desynchronization recorded over areas related to right-hand movement (left parietal cluster) did not differentiate research groups. The right and left-handers shared a similar degree of lateralization of the ERD effect during KMI and VMI conditions. It should be noted that the signal in both parietal clusters showed higher desynchronization during imagery movement for the contralateral hand. Similar findings were reported in the article of Zapała et al.^19^ where differences between the right and left-handers in alpha suppression were observed only during left hand MI in contralateral ICs cluster. Intergroup differences in alpha and beta topography are also visible on the signal distribution maps on the skull (Supplementary Fig. 4). It can be seen that right-handers show characteristic lateralization of SMR suppression, regardless of the experimental condition. In the second group, less intensive ERD lateralization occurs mainly in the KMI condition. Probably it means that only left-handers changed their imagery perspective according to the experimental instruction. In some studies (e.g.^3,26^), lack of clear SMR lateralization patterns was associated with MI's performance in a non-kinaesthetic way. On the other hand, as Bai et al.^27^ suggest, the ERD's less pronounced lateralization may indicate greater complexity of the motor task or the need to inhibit the other hand's movement. It seems probable that left-handers had to put more effort into the "feel" of the non-dominant hand movement's kinaesthetic sensation, which triggered a bilateral activation.

The electrophysiological results confirm some previous findings regarding the relationship between handedness and asymmetry of brain activity. Less asymmetry of SMR distribution in left-handers recorded in parietal clusters corresponds to other EEG^19^, fMRI^16^ and TMS^15^ studies. Similar bilateral lateralization in left-handers has also been observed concerning the cerebral representation of spatial functions. According to meta-analyses^28,29^, in right-handers, the areas associated with the spatial task are located in the right hemisphere, while no hemispheric preference was found in left-handers. However, at the behavioural level, the left-handers' MI performance is better than right-handers, which is opposite to the results of studies where the MI perspective was not considered. It is possible that in tasks without explicit instruction for MI perspective (such as HLT^14^ or the *Graz BCI*^19^) left-handers use default strategies (e.g. VMI) that do not produce optimal results in terms of time^14^ or accuracy^19^. In the present experiment, this effect did not occur because participants were asked to create a specific movement representation. Another result that seems to be confirmed is the deactivation of the visual cortex, which has been linked to concentration on internal states in other studies^5^. It might explain why better VMI performance in left-handers accompanied synchronization of the occipital alpha band.

Our results may have significant implications for the development of MI-BCIs. Left-handed people represent a large (approx. 10–13% humans)^30^ and heterogeneous^31^ part of the population of potential BCI users. Current studies focus mainly on the right-handed population^32^, so new solutions in terms of, e.g. signal analysis or user training should be proposed based on the characteristics of this group. Consequently, there is a risk of a mismatch between the developed technology and the neurophysiological (ERD-SMR lateralisation) and cognitive (preference in MI perspective) diversity of users^32^. Indeed, there is much evidence of asymmetry in brain function^33^, behaviour and cognitive activity^34^, and the effectiveness of BCI communication relies on all of these mechanisms^35^. Moreover, one of the main target groups for brain-computer interfaces are people suffering from brain damage (e.g. after stroke)^36^ or developing neurodegenerative diseases^37^. In both cases, asymmetric brain activity changes can occur^38,39^, so this aspect also deserves more attention^40^.

Some limitations of these results should also be noted. First of all, the imagery perspectives were only evoked by the preceding block's execution (ME and AO) and explicit instruction. There are no objective or subjective indicators of perspective change by the participants. It is possible that subjects used their preferred perspective regardless of the instructions in the block. For example, if it was more effective for someone to imagine movement in a kinesthetic form, they could refer to these sensations in the VMI condition. Therefore, the procedure used does not guarantee that the obtained results are not due to differences in following instructions between experimental groups.

Moreover, AO and ME conditions were not included in the EEG data analysis because they differed from the MI conditions in terms of visual stimulation (AO) and motion activity (ME). It is also likely that the differences in brain activity patterns may have been present in other frequency ranges (e.g., low and high alpha^41,42^) and locations (e.g. frontal areas^43^) than those included in the hypotheses. The results of dipole localization should be treated with great caution as they are only approximate estimates of signal sources in the brain. Also, the performance of MI tasks with eyes closed and auditory cues would prevent potential overlap processing of visual stimuli and mental images. Finally, handedness can be considered in a more complex way than the applied dichotomous classification^44^.

## Conclusions

Our studies' results may provide new explanations of the different effects of the research on the relationship between handedness and MI-BCIs control^18,19^. According to our hypotheses, right-and left-handed differ in their ability to imagine a movement from a kinesthetic and visual-motor perspective. We have obtained partial confirmation of these assumptions at the behavioural and electroencephalographic levels. However, contrary to expectations, left-handers have achieved better results in the MI task, regardless of the experimental condition. It may result from the life experience of this group, who is forced to change their perspective more frequently in order to be able to use the tools designed with the right-handed. Another possible explanation is the reported differences in neuroanatomy^45^ and connections between cerebral structures^16^. However, due to limitations in our research procedure, this issue should be addressed in future studies.

## Methods

### Participants

40 subjects (35 females) aged 20–41 years (*M* = 24.70; *SD* = 4.56) participated in the experiment. The left-handed group consisted of 20 subjects (17 females; aged 20–40 years; *M* = 24.20; *SD* = 4.80) and the right-handed group consisted of 20 subjects (18 females; aged 21–40 years; *M* = 25.10; *SD* = 4.40). All subjects performed the Edinburgh Handedness Inventory (EHI), which assesses hand dominance^46^ and the Movement Imagery Questionnaire-Revised Second Version (MIQ-RS)^47^ to control motor imagery ability. To determine whether the groups demonstrate the dominance of the left/right hand and vividness of motor imagery at a similar level, the Mann–Whitney *U* test was conducted. The non-parametric test was applied because the MIQ results in the left-handed group and the EHI in both groups have not met the assumption of normality (*p* < 0.05 in the Shapiro–Wilk test).

In both the left-handed (*Me* = 83.5; *Q* = 17.5) and the right-handed (*Me* = 87; *Q* = 10) group the score of the EHI was high and the results did not differ between groups (*U* = 166.50; *p* = 0.348). Motor imagery was calculated separately for: visual motor imagery—VMI_left-handed_ (*Me* = 6; *Q* = 0.71); VMI_right-handed_ (*Me* = 5.78; *Q* = 0.79) and kinesthetic motor imagery—KMI_left-handed_ (*Me* = 6; *Q* = 0.28); KMI_right-handed_ (*Me* = 5.64; *Q* = 0.64). Both groups were not significantly different in terms of: VMI (*U* = 158; *p* = 0.254) or KMI (*U* = 199.50; *p* = 0.989).

Written information consent was obtained from all participants who took part in the experiment. They also declared that they were neither taking medication nor any psychoactive substances permanently. The study was conducted in compliance with the Declaration of Helsinki and approved by the Ethics Committee of the Institute of Psychology at the John Paul II Catholic University of Lublin.

### Apparatus

The brain's electrical activity was measured with a GES 300 (Electrical Geodesics, Inc. Eugene, OR, USA) EEG system, comprising a Net Amps 300 amplifier and a 64-channel actiCAP (Brain Products, Munich, Germany) cap with active electrodes. Electrode impedances were kept below 10 kΩ, and the signal was referenced to an FCz channel during registration. Data sampling was defined at 500 Hz and recorded with a Net Station 4.4 (EGI, Eugene, OR, USA).

**Figure 5:**
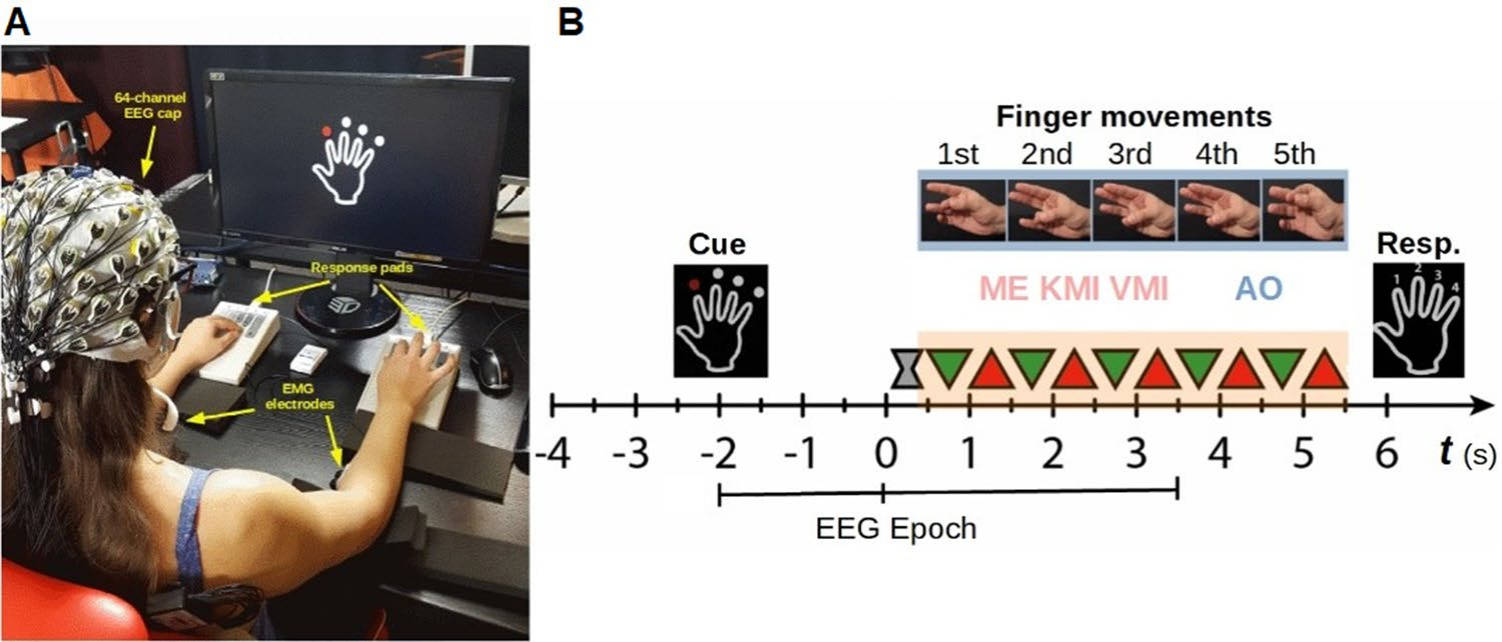
(**A**) Experimental setup and (**B**) procedure. In each block, participants were instructed to observe (AO), execute (ME), or imagine (KMI, VMI) fingers movements of their right or left hand, starting from a finger which was indicated with a red dot. Then the gray hourglass symbol was displayed to indicate the time for preparing to begin the task. During finger movements, a green triangle was presented in the middle of the screen as a signal to contact the fingers and a red triangle as a signal to distance them (ME, KMI, and VMI conditions). In the AO condition, a video sequence with recorded finger movement was displayed instead of triangles. Each trial was composed of three to five movements (randomly). At the end of a sequence, participants were asked to press the button corresponding to the last finger movement. The trial structure was a modified version of the procedure used by Kim et al.^48^. Used software to create the images in this figure: LibreOffice Draw (The Document Foundation, Berlin, Germany, https://www.libreoffice.org/discover/draw/).

The experimental procedure was designed and displayed on a screen using E-Prime, version 2.0.10.356 (Psychology Software Tools, Pittsburgh, PA, USA). Visual stimuli were displayed on a 23 in. LCD monitor (Dell, Inc., Round Rock, TX, USA) screen with a resolution of 1920  ×  1080 pixels and a refresh rate of 60 Hz. The monitor was located at a distance of 70 cm from the subject. The EEG signal acquisition and stimuli presentation were performed using apparatus settings from a similar study by Zapała et al.^19^. Subjects answered using two 5-button custom made response pads (see^41^), one for the left and one for the right hand (Fig. 5A).

Electromyographic (EMG) measurements were carried out during the EEG registration to control the hands' inadvertent movements during experimental conditions. Two surface T3402 Triode electrodes were attached to the *flexor digitorum profundus* of both arms and recorded using a Flex Comp Infinity system (Thought Technology Ltd., Montreal, Canada).

Signal processing of the EEG records was performed using EEGLab 14.0.0b, a toolbox to MATLAB (MathWorks, Natick, MA, USA). Statistical analysis of the results was conducted with STATISTICA 12 (StatSoft, Inc., Tulsa, OK, USA) software.

### Procedure

The procedure consisted of two parts: (1) the training protocol, which involved fingers movements practice with EMG feedback presentation, and (2) the experimental session consisted of EEG measurements during tested conditions.

The training protocol was adopted from the studies of Stinear et al.^6^. The subjects were asked to synchronize their fingers' movement with the type of clue presented on the screen. Similar to the study by Burianová et al.^22^ the fingers of each hand were assigned numbers (1 = thumb, 2 = index finger, 3 = middle finger, 4 = ring finger, 5 = little finger) and participants were asked to perform/observe/imagine the same sequence in each trial (2–1; 2–1; 3–1; 4–1; 4–1) starting from the finger pointed by the visual cue (Fig. 5B). All conditions were described to the subjects during training and presented again as a written instruction during the experimental session. The Motor execution (ME) condition instructions were to “perform a sequential movement of your fingers in time of the clues displayed on the screen”. The Kinesthetic Motor Imagery (KMI) condition instructions were to “imagine a sequential movement of your fingers in time of the clues displayed on the screen, and the feeling that this produces” The Action observation (AO) condition instructions were to “observe carefully the movement of the fingers displayed on the screen”. The Visual Motor Imagery (VMI) condition instructions were to “imagine seeing sequential movement of your fingers in time of the clues displayed on the screen”. Additionally, the participants were asked to observe and imagine the performance of this task without real movement. In AO, KMI, and VMI training rehearsals, participants heard acoustic feedback every time when muscle tension above a set threshold was detected.

The experiment session was divided into two blocks where the sequence of the conditions was set to facilitate a change of the motor imagery perspective, similar to the study by Neuper et al.^3^. One of the blocks began with the ME and then KMI was performed, the other started with the AO and then the VMI was presented. The order of the blocks was counterbalanced between subjects. Each block consisted of an introduction, series of testing (n = 8), and practice trials (n = 48). During the testing trials, participants received feedback on whether the button they press corresponded to the correct finger in the movements' sequence. When they reached at least five correct answers, they started the practice trials with EEG registration. In the experimental session, each subject performed 192 practice trials (48 trials in four TASK and two HAND conditions).

### Data processing

Behavioral data were recorded automatically in the experiment control software. Statistical analyses compared the accurate responses, i.e., the number of trials where a participant pressed the button corresponding to the correct finger in a sequence of movements.

The EEG signal was bandpass filtered in the 1–40 Hz range using finite impulse response filter (FIR filter) and subjected to re-referencing (CAR, common average reference). Short-time high-amplitude artifacts have been removed by the Artifact Subspace Reconstruction (ASR) method as implemented in Clean Raw Data plug-in^49,50^. Then, the ICA decomposition and equivalent current dipole model were conducted using *runnica* algorithm and boundary element head model (BEM) using DIPFIT plug-in^51^. The ICs that may represent eye blinking, lateral eye movement, muscle activity, electrical noise, with a residual variance of dipole higher than 15% or located outside the brain, were removed from further analysis using ICLabel plug-in^52^. The signal was divided into 5500 ms long segments (baseline from − 2000 to 0 ms, event period from 0 to 3500 ms) (Fig. 2B) and subjected to time–frequency decomposition with Event-Related Spectral Perturbation^53^ using sinusoidal wavelet transformations (3-cycles; 0.5 s) to calculate the signal strength (dB) for the entire window. For statistical analyses, signal strength for alpha (8–13 Hz) and beta (15–30 Hz) frequency bands in the same time window (500–3000 ms) was averaged across individual ICs within the parietal and occipital clusters. The statistical analysis was carried out separately for three ICs clusters: parietal right, parietal left (Figs. 3B and 4B) and central occipital (Fig. 2B).

To verify all research hypotheses a mixed design ANOVAs was conducted with the between-subject factor Group (left-handed vs. right-handed), and 2 × 2 within-subjects factors: Task (KMI/VMI) and Hand (Left/Right), separately for behavioral and electroencephalographic data. Moreover, the ERSPs distribution maps were computed to illustrate the differences in brain wave topography (Supplementary Fig. 4).

The root mean squared amplitude (mV) of EMG signal from the right and left forearm were calculated for the entire EEG event period (0–3000 ms) and a repeated-measures ANOVA was used with the between-subject factor Group (left-handed vs. right-handed), and 2 × 2 × 2 within-subjects factors: Task (KMI/VMI), Hand (Left/Right) and Channel (Left/Right).

## Supplementary Information

Supplementary Information.
