# Supplementary material for: Handedness effects on motor imagery during kinesthetic and visual-motor conditions
Source: Sci Rep. 2021 Jun 23;11:13112. doi: 10.1038/s41598-021-92467-7 (PMC8222290; doi:10.1038/s41598-021-92467-7)
Supplement: Supplementary file 1 — Supplementary Information. [file 41598_2021_92467_MOESM1_ESM.pdf]

Handedness effects on motor imagery during kinesthetic and visual-motor conditions

Dariusz Zapała, Paulina Iwanowicz, Piotr Francuz and Paweł Augustynowicz

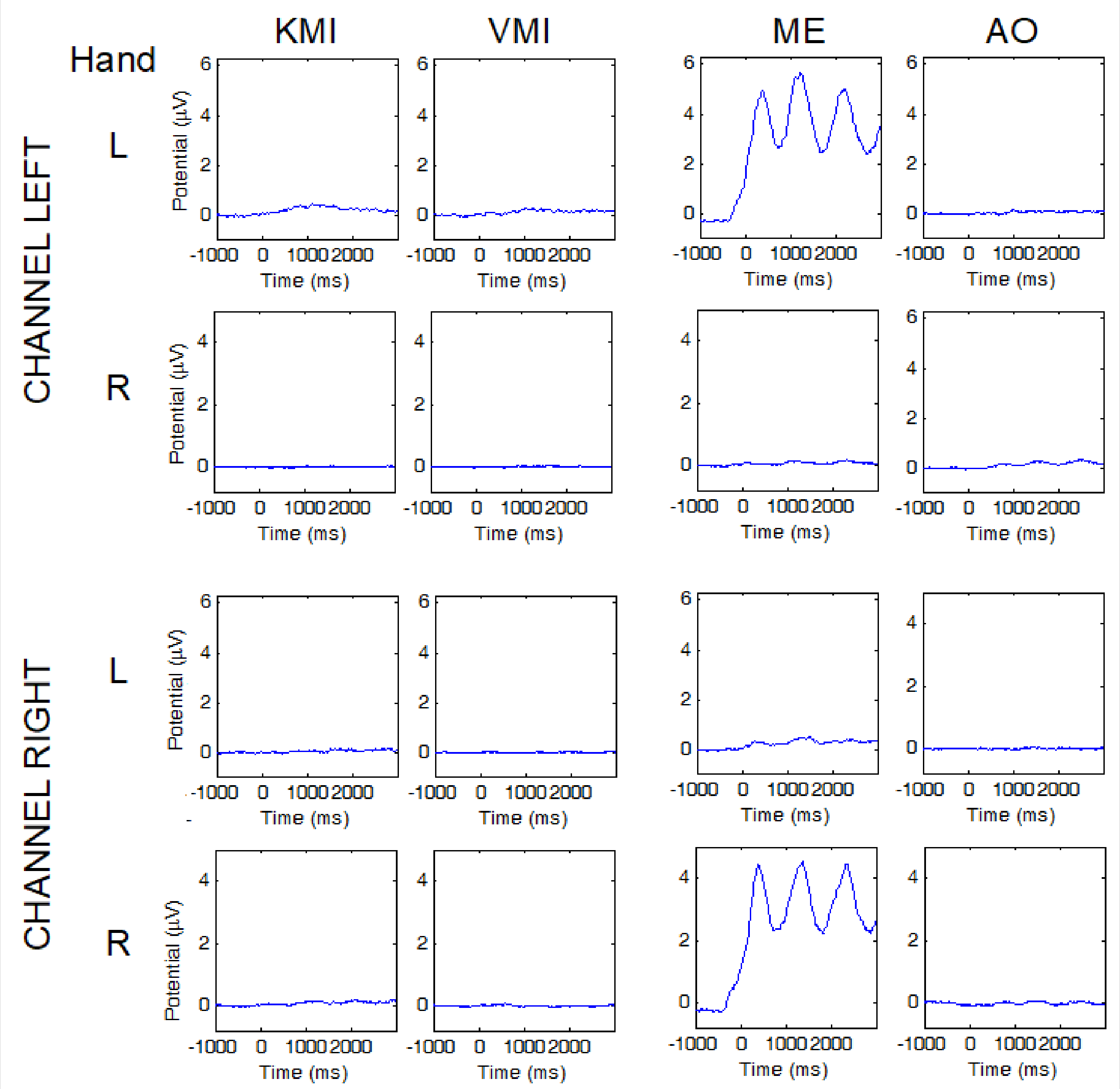

Supplementary Figure 1. Average RMS EMG amplitude for both experimental (left column) and execute/observation conditions (right column). Used software to create the images in this figure: MATLAB version R2014b (MathWorks, Natick, MA, USA, https://www.mathworks.com/).

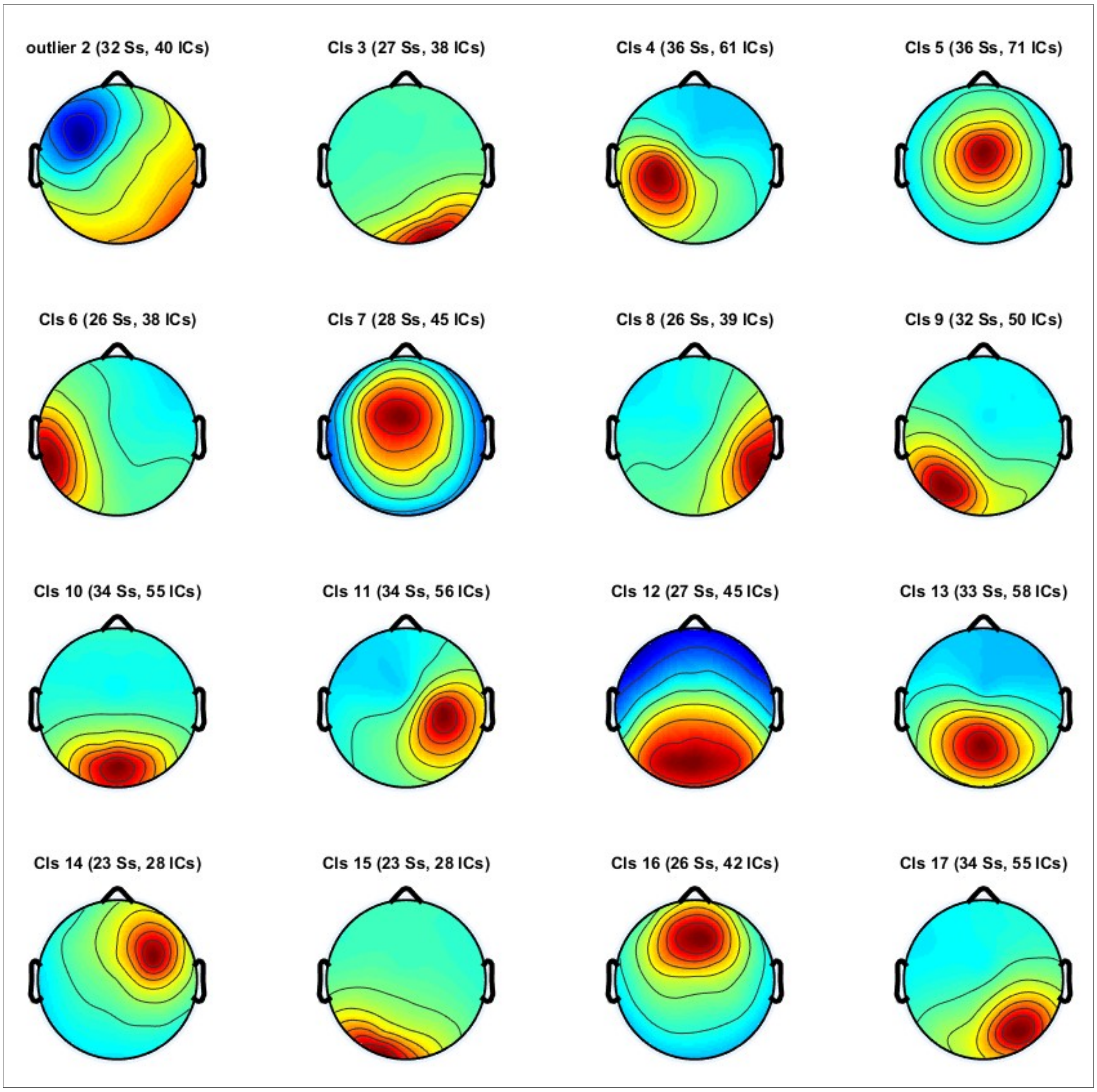

Supplementary Figure 2. Average scalp maps for all clusters. Ss – number of subjects; ICs – number of sources. Used software to create the images in this figure: MATLAB version R2014b (MathWorks, Natick, MA, USA, https://www.mathworks.com/).

Supplementary Table 1. Description and measurement of the study variables.

Variable	Description	Measurement
Independent	Group	Left-handed; Right-handed
Independent	Task	KMI; VMI; ME; AO
Independent	Hand	Left hand; Right hand
Dependent	Performance	The number of accurate responses
Dependent	EEG (signal strength)	The ERSP signal strength (dB) for alpha (8 - 13 Hz) and beta (15 - 30 Hz) frequency bands in the time window from 500 - 3000 ms, averaged across individual ICs within the motor-related (left and right parietal) and visual-related (central occipital) clusters
Dependent	EEG (distribution)	The ERSP signal distribution on the skull (8-13 Hz)
Control	EMG	Average RMS amplitude from left (channel-left) and right (channel-right) forearm
Control	MI ability	KMI scale; VMI scale
Control	Hand dominance	Laterality Quotient

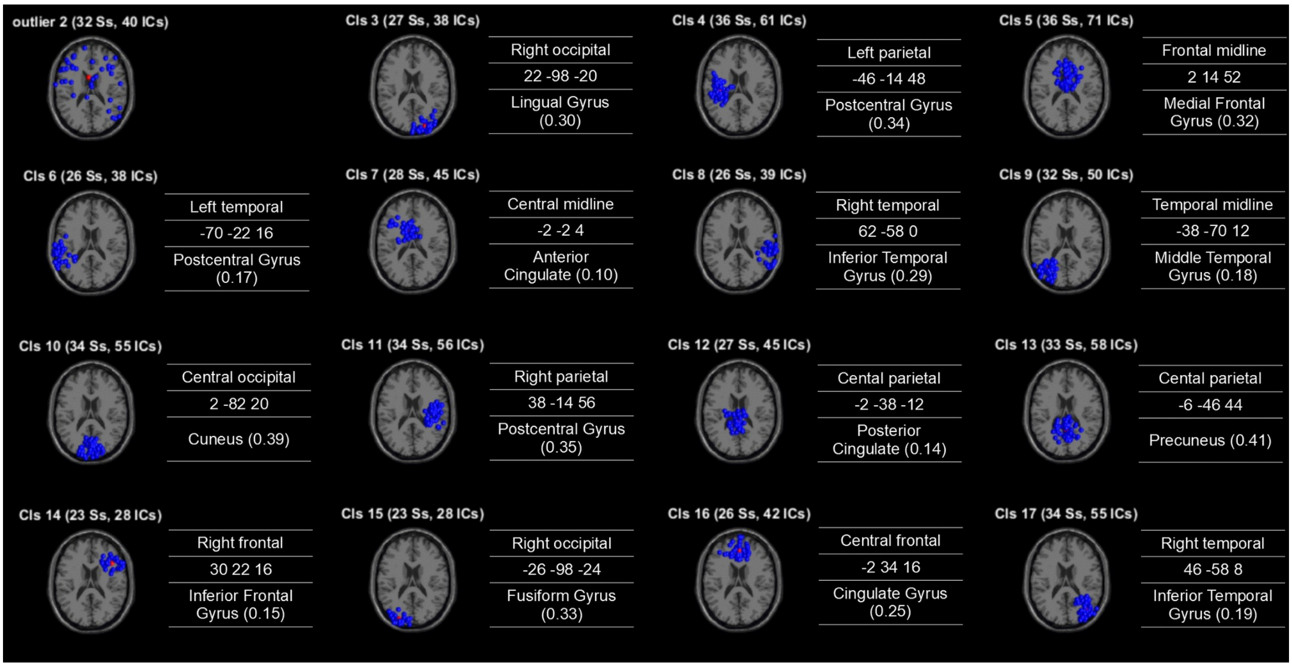

Supplementary Figure 3. Estimated dipole locations of ICs (*blue dots*) with the MNI coordinates of cluster centroid and the probabilistic position in anatomical areas (*table*). Used software to create the images in this figure: MATLAB version R2014b (MathWorks, Natick, MA, USA, https://www.mathworks.com/).

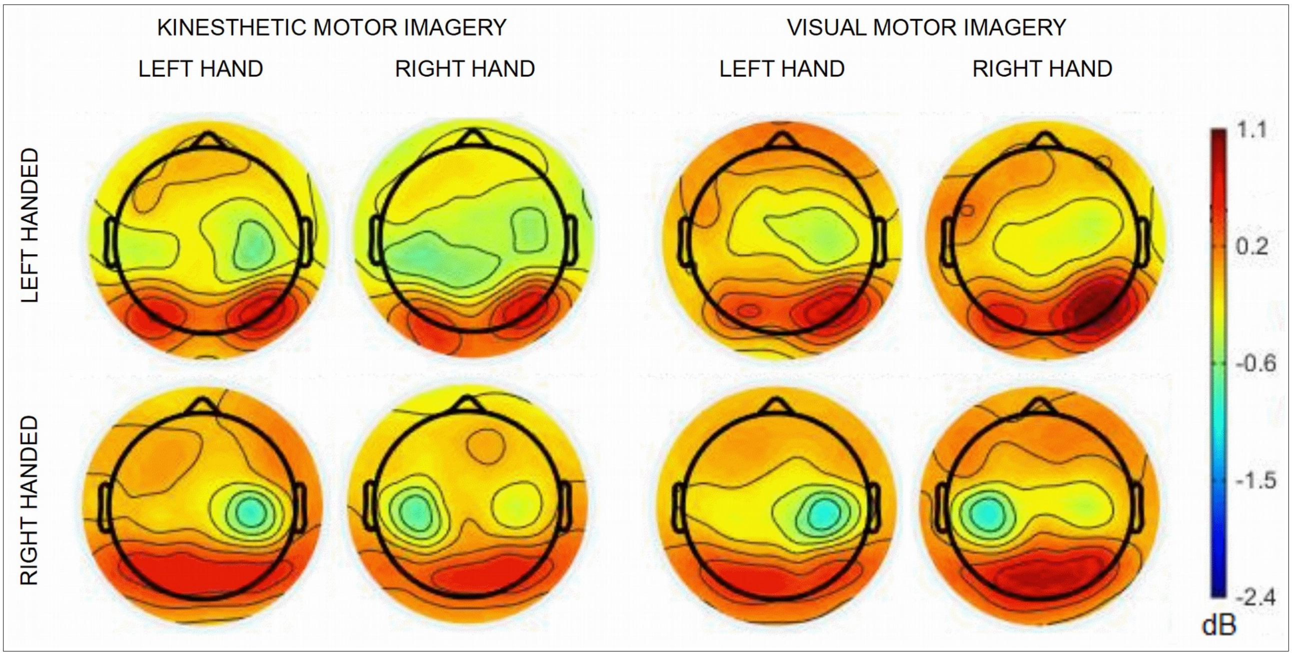

Supplementary Figure 4. Maps of ERSP distribution on the skull (8–30 Hz) during the imagined movement of the right and left hand in the KMI or VMI conditions. Used software to create the images in this figure: MATLAB version R2014b (MathWorks, Natick, MA, USA, https://www.mathworks.com/).
